# Effectiveness, safety and cost reduction of long-term tunneled central venous catheter insertion in outpatients performed by an interventional nephrologist

**DOI:** 10.1590/2175-8239-JBN-2019-0108

**Published:** 2019-10-24

**Authors:** Artur Quintiliano, Marcel Rodrigues Gurgel Praxedes

**Affiliations:** 1 Universidade Federal do Rio Grande do Norte, Departamento de Medicina Integrada, Natal, RN, Brasil.; 2 Hospital Monsenhor Walfredo Gurgel, Natal, RN, Brasil.

**Keywords:** Vascular Access Devices, Renal Replacement Therapy, Cost Savings, Dispositivos de Acesso Vascular, Redução de Custos, Terapia de Substituição Renal

## Abstract

**Introduction:**

Invasive procedures performed by trained nephrologists can reduce delays in making a definitive vascular access, complications, number of procedures on the same patient, and costs for the Public Health System.

**Objective:**

to demonstrate that a long-term tunneled central venous catheter (LTCVC) implanted by a nephrologist is safe, effective, and associated with excellent results.

**Methods:**

A retrospective study analyzed 149 consecutively performed temporary-to-long-term tunneled central venous catheter conversions in the operating room (OR) from a dialysis facility from March 2014 to September 2017. The data collected consisted of the total procedures performed, demographic characteristics of the study population, rates of success, aborted procedure, failure, complications, and catheter survival, and costs.

**Results:**

the main causes of end stage renal disease (ESRD) were systemic arterial hypertension and diabetes mellitus, 37.9% each. Patients had a high number of previous arteriovenous fistula (1.72 ± 0.84) and temporary catheter (2.87 ± 1.9) attempts until a definitive vascular access was achieved, while the preferred vascular site was right internal jugular vein (80%). Success, abortion, and failure rates were 93.3%, 2.7% and 4%, respectively, with only 5.36% of complications (minors). Overall LTCVC survival rates over 1, 3, 6, and 12 months were 93.38, 71.81, 54.36, and 30.2%, respectively, with a mean of 298 ± 280 days (median 198 days). The procedure cost was around 496 dollars. Catheter dysfunction was the main reason for catheter removal (34%).

**Conclusion:**

Our analysis shows that placement of LTCVC by a nephrologist in an OR of a dialysis center is effective, safe, and results in substantial cost savings.

## Resumo

**Introdução:**

Procedimentos invasivos realizados por nefrologistas podem reduzir o número de procedimentos no mesmo paciente, complicações e atrasos na obtenção de acesso vascular definitivo, bem como proporcionar menor custo para o Sistema de Saúde.

**Objetivo:**

Demonstrar a segurança, a eficácia e os resultados dos implantes de cateteres venosos centrais de longa permanência (CLP) realizados por nefrologista sem fluoroscopia.

**Métodos:**

Estudo retrospectivo que analisou 149 implantes de CLP por nefrologista no centro cirúrgico de clínica de diálise, sem auxílio de fluoroscopia, no período de março/2014 a setembro/2017. Os dados coletados consistiram em: características demográficas da população estudada, taxas de sucesso, procedimento abortado, falha no procedimento, complicações observadas, patência do cateter e custos.

**Resultados:**

Houve um elevado número de tentativas fístulas arteriovenosas (1,72 ± 0,84) e de cateter de curta permanência (2,87 ± 1,9) até a realização de um acesso vascular definitivo. O sítio vascular preferido foi a veia jugular interna direita (80%). Taxas de sucesso, procedimentos abortados e falhas foram de 93,3%, 2,7% e 4,0%, respectivamente, com apenas 5,36% de pequenas complicações. A patência dos CLP com 1, 3, 6 e 12 meses foram de 93,38%, 71,81, 54,36% e 30,2%, respectivamente, com média de 298 ± 280 dias (mediana 198 dias). Os custos dos procedimentos foram em torno de US$ 496. Disfunção foi o principal motivo da remoção do cateter (34%).

**Conclusão:**

Nossa análise mostra que o implante de CLP por nefrologista no centro cirúrgico de clínica de diálise é eficaz e seguro e está associado à redução significativa de custos.

## Introduction

The vascular access for hemodialysis treatment is better achieved with the use of an arteriovenous fistula (AVF). However, catheters for hemodialysis are widely used in current practice: 15-50% of patients in Europe and 60% in the United States had their treatment started with a catheter as primary access^1^. The updated National Kidney Foundation Kidney Disease Outcomes Quality Initiative (KDOQI) guidelines recommend that when a temporary catheter (TC) will be needed for more than three weeks, a *long*-*term tunneled central venous catheter* (LTCVC) should be used^2^.

Invasive procedures performed by a nephrologist instead of a vascular surgeon or interventional radiologist, especially in areas of difficult access, can reduce delays in making a definitive vascular access, complications, number of procedures on the same patient, and costs for Public Health System. Therefore, nephrologists have taken the initiative to perform these proceedings themselves. Indeed, recent data have shown that nephrologists can safely and successfully perform these procedures with excellent results^3^.

In this study, we assessed the effectiveness and safety of a TC conversion to a LTCVC performed by a nephrologist without using fluoroscopy in an outpatient center. We also verified the clinical outcomes, complications, and cost of the procedure.

## Methodology

This was a retrospective study of incident and non-incident dialytic patients. We performed 149 temporary-to-*long-term tunneled central venous catheter* conversions in the operating room of a dialysis facility from March 2014 to September 2017, in Natal - RN (Brazil). Catheters were placed in the internal jugular or femoral veins. Prophylactic antibiotic was not routinely administered. Reasons for catheter removal were creation of an arteriovenous fistula, transfer to another dialysis modality, recovery of renal function, kidney transplant, death, infection, dysfunction, and accidental removal.

Data on all inserted catheters, clinical follow-up, and catheter-related complications were recorded by the nephrologist and by the dialysis nursing team. The data collected consisted of the total procedures performed, demographic characteristics, rates of success, aborted procedure, failure, complications and catheter survival, and costs.

### Techniques of long-term tunneled central venous catheter insertion (figure 1)

**Figure 1:**
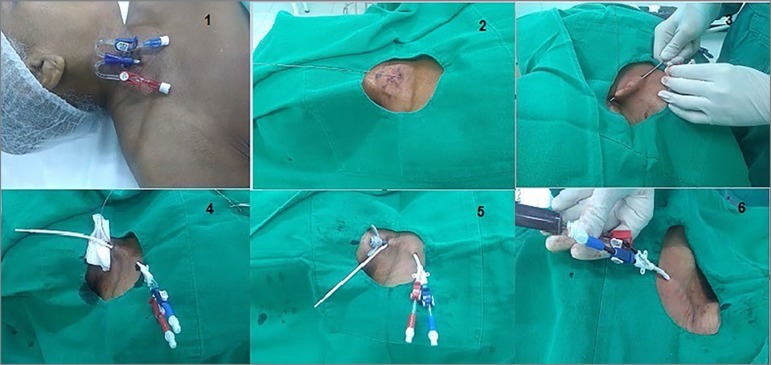
Sequence of the catheter placement. 1) Temporary catheter (TC) in right internal jugular vein (RIJV), 2-3 cm above the clavicle; 2) Introduction of guidewire and removal of TC; 3) Creation of the subcutaneous tunnel by a dilator-catheter; 4) Passage of the longterm tunneled central venous catheter (LTCVC) through the tunnel ; 5) Introduction of the "peel-way" sheath by the guidewire and removal of the guidewire; 6) LTCVC placed.

All procedures were performed by the same nephrologist and a nurse technician in an operating room of the dialysis facility and under sterile conditions, without fluoroscopy assistance, and guided by ultrasound when necessary. Preferred vascular site for implantation, in order of preference, were right internal jugular vein (RIJV), left internal jugular vein (LIJV), and femoral veins (FV). Whenever the LTCVC was in a FV, a new puncture was first attempted in the RIJV, because this site was admittedly a better option. If success was not achieved, the definitive catheter implant was maintained in this site.

As there was no fluoroscopic assistance, the length of the tunneled catheter inside the vein was based on the chest X-ray performed before conversion, so that the tip of the LTCVC entered the right atrium (5 cm below the mainstream bronchus). If the insertion site of was too high (3 cm from the clavicle), patients were submitted to a *de novo long-term tunneled central venous catheter* placement nearest from the clavicle. This maneuver aims to ensure a smooth curvature and prevent kinking (i.e. focal buckling) of the LTCVC. In all patients in our sample were used tip dual lumen catheter (MEDCOMP Inc., Harleysville, PA, USA), 14.5 French diameter, with an overall and implant cuff-to-tip length ranging from 36-45 and 19-35 cm, respectively, depending on the insertion site, tunnel extension, and patient size. Sedation was not necessary, only local anesthesia with lidocaine 2%.

TC was converted to LTCVC by advancing a guidewire through one of the venous lumen of the existing temporary catheter, which was then removed. Afterward, a subcutaneous tunnel was created and the catheter passed through the tunnel using the tunneler provided with the catheter kit. The next step consisted of placing a peel-away sheath/dilator combination over the guidewire. The dilator and wire were removed and the catheter was inserted centrally through the sheath, which was peeled away.

1) Temporary catheter (TC) in right internal jugular vein (RIJV), 2-3 cm above the clavicle; 2) Introduction of guidewire and removal of TC; 3) Creation of the subcutaneous tunnel by a dilator-catheter; 4) Passage of the *long-term tunneled central venous catheter* (LTCVC) through the tunnel ; 5) Introduction of the “peel-way” sheath by the guidewire and removal of the guidewire; 6) LTCVC placed.

### Catheter removal

Indications for catheter removal were fistula creation, transfer of therapy modality, recovery of renal function, kidney transplant, death, infection, dysfunction^2^ (failure to attain or maintain an extracorporeal blood flow > 300 mL/min at a prepump arterial pressure lower than −250 mm Hg during the first 60 minutes of hemodialysis), and accidental removal. The catheters were removed without fluoroscopic guidance or sedation, only with local anesthesia. Guide-wire was used when there was a replacement intention of a LTCVC for a short-term catheter or other *long-term tunneled central venous catheter*, situation where a new exit site and tunnel were created.

### Management during the procedure

Blood pressure, heart rate, and oxygen saturation were monitored continuously during the performance of all procedures by a nurse technician.

### Outcomes of procedures

The result for each procedure performed was recorded as a success, failure, or aborted. The procedure was classified as successful if the catheter achieve sufficient blood flow to perform a single hemodialysis treatment and this information was reported by the dialysis facility. Blood flow was set to 300 mL/ min or greater^3^. Failure was defined if the process was completed but without fulfilling the established criterion for success. A procedure was considered aborted when it was attempted, but could not be completed and abandoned. Both failure and aborted should be considered unsuccessful procedures.

## Complications

The complications observed in this study were classified according of the Society of Interventional Radiology^4^. According to this rule, all the complications, including pulmonary and cardiac events, occurring within 30 days after the procedure are considered related to the procedure. Minor complications are those that require no specific therapy and are resolved without any adverse consequence. The major complications are defined as those that require an increase in the level of care or result in hospitalization, permanent adverse sequelae, or death. If a complication cannot be successfully treated, it results in failure of the procedure. Late bleeding was defined as a bleeding episode requiring medical management after initial hemostasis had been achieved. Hypotension (SBP ≤ 90 mm Hg or DBP ≤ 60 mmHg) and oxygen saturation ≤ 90% at any time during the procedure were considered complications.

### Costs included and excluded from the analyses

Physician fees for anesthetist, surgeons and radiologists, operating and recovery room time and nursing, fluoroscopy assistance, and anesthesia were included in this study because the purpose was to determine the difference in costs from the perspective of the center analyzed. Excluded from the analysis were any tests to evaluate a patient’s underlying medical problem or complication that might have occurred on the same day as catheter placement.

### Statistical analysis

Data are described as frequency distribution and mean and median, as appropriate. Categorical variables were expressed as frequency and percentages. Survival analysis was performed using the Kaplan-Meier method. All statistical analysis was performed using IBM SPSS^®^ 22 Statistics software.

## Results

Patients were predominantly female (52.63%), were in the fifth decade of life (55 ± 15.1 years) and the main causes of ESRD were systemic arterial hypertension (SAH) and diabetes mellitus (DM), 37.58% each one, followed by chronic glomerulonephritis - GN (13.42%). The main comorbidities of the patients were SAH (77.85%), DM (37.58%), heart disease (8.72%), and obesity (4.49%). Patients had a high number of previous AVF (1.72 ± 0.84) and TC (2.87 ± 1.9) attempts until a definitive vascular access was achieved, with the preferred vascular sites being RIJV (79.86%), FV (11.40%), and LIJV (6.05%). Twelve patients had an even higher number of catheters, with 8 (n = 7 patients), 9 (n = 5 patients), and 9 (n = 1 patient) TCs. Time from the first TC placement until TC- LTCVC conversion was 4.2 ± 1.07 months (Table 1).

**Table 1:** Demographic characteristics

Variable	n = 149
Age (years)	55 ± 15.1
Gender (Female - %)	52.63%
Etiology of CKD (n - %)	
Hypertension	56 - 37.58%
Diabetes mellitus	56 - 37.58%
Chronic glomerulonephritis	20 - 13.42%
Others	17 - 11.40%
Comorbidades (n - %)	
Hypertension	116 - 77.85%
Diabetes mellitus	56 - 37.58%
Cardiac failure congestive	8 - 8.72%
Obesity	7 - 4.49%
Previous TP (mean ± SD)	2.87 ± 1.9
Previous AV fistula (mean ± SD)	1.72 ± 0.84
Sítio vascular (n - %)	
Right internal jugular vein	119 - 79.86%
Femoral veins	17 - 11.40%
Left internal jugular vein	9 - 6.04%
Left subclavian vein	3 - 2.01%
Right subclavian vein	1 - 0.67%

Nota: DRC - doença renal crônica; FAV - fístula arteriovenosa; CCP - cateter de curta permanência; DP - desvio-padrão.

A total of 139 (93.3%) cases were successful, with only 4 (2.7%) cases aborted, 1 by excessive agitation of the patient and 3 by failure to perform TC-to- LTCVC conversion. The procedure failed (the catheter was removed) in only 6 (4%) patients due to the catheter not showing blood flow. A small number of patients had minor bleeding (n = 7, 4.7%) and none had a major bleeding or needed a blood transfusion. The main complications (n = 8, 5.36%) were mild hypotension (n = 6, 4.02%) and desaturation (n = 2, 1.34%), with no death, hemodynamic instability, arrhythmias, respiratory failure occurring during or immediately after the procedures or any hospitalizations in the first 30 days after the procedures (Table 2).

**Table 2:** Outcomes of procedures

Variável	n - %
Success (n - %)	139 - 93.3%
Abortaded (n - %)	4 - 2.70%
Failure (n - %)	6 - 4.00%
Minor bleeding	7 - 4.70%
Major bleeding	0 - 0 %
Complications (n - %)	8 - 5.36%
Hypotension	6 - 4.02%
Hypoxemia	2 - 1.34%
Death	0 - 0%
Arrhythmia/HI/RF	0 - 0%

HI: hemodynamic instability; RF: respiratory failure.

The total cost of each procedure was around $ 496 dollars (placement), of which $ 460 (catheter included) were for fixed direct costs and $ 36 for variable costs. 

**Figure 2:**
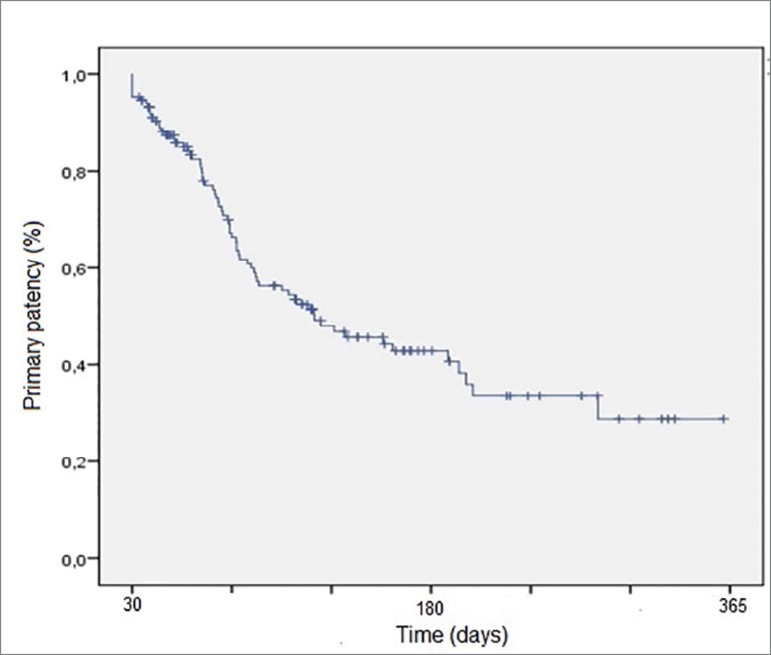
Catheters patency.

**Figure 3:**
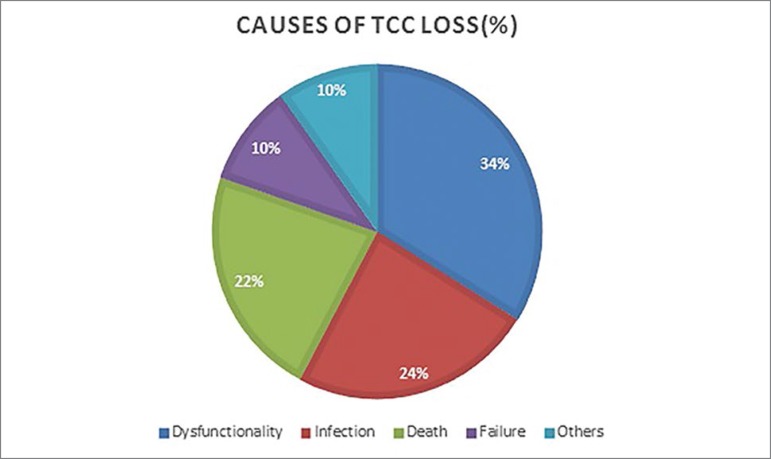
Causes of LTCVC loss.

Overall LTCVC survival rates over 1, 3, 6, and 12 months were 93.38, 71.81, 54.36, and 30.2%, respectively, with a mean of 298 ± 280 days (median 198 days) - Figure 2. The main causes of catheter removal (n = 71) were dysfunctionality (n = 24, 33.8%), infection (n = 17, 23.94%), death (n = 16, 22.5%), unsuccessful procedure (n = 7, 9.88%) and transplantation, therapy modality modification, discharge of the patient from dialysis facility, or performance of AVF (n = 7, 9.88%) - Figure 3. The mean of the first infection occurred was 8.14 ± 6.4 months, infection rate was 1.87/1000 catheter-day, and infections requiring catheter removal was 0.7/1000 catheter-day. Considering the period of 30 days after catheter implant, there was only 1.34% of infections (two patients), related to the implant procedure.

## Discussion

All patients of this study started hemodialysis through TC and the overwhelming majority of them had an elevated number of AVF attempts or catheter exchanges until the placement of the LTCVC. This was due to primary failure of the newly created hemodialysis AV fistula secondary to the high number of diabetic and elderly patients, late failure of a hemodialysis AVF (vascular stenotic lesions), multiple infections of catheter, catheter initially implanted in femoral veins and catheter dysfunction. Additionally, the duration of time required for conversion from TC to LTCVC in dialysis patients was prolonged (4 months).

The technique proposed by our study showed to be safe and effective, even though it was performed in the operating room of a dialysis clinic, with only eight (5.3%) cases of mild complications and a success rate of 93.3%. Our complication rates are in agreement with other publications^5^^,^ 6, which have shown that this type of procedure is safe. The patency showed in our study was 91.94, 82.5, and 63 in 30, 60, and 120 days, respectively, while another study^7^ demonstrated a success rate of 100% in placements, but with procedures performed in the hospital regime, and patency was 92, 82, and 68 for the same period. We had an early failure (< 30 days) that required catheter removal of 6.62%, superior of that reported in the literature - 1.4%^8^.

The major cause of catheter removal was dysfunctionality, probably due to thrombosis, fibrin sheath formation at the catheter tip, malpositioned catheter tip, and kinking. As the procedures were done without fluoroscopy assistance, there may have been kinks (depending on flexion and angle degree) that could not be detected or corrected at the time of catheter placement. Thus, the best ways to optimize catheter flow were to reduce kinks by puncturing the jugular vein as close as possible to the clavicle, and a well-planned approach for the tip of the catheter to be in the right atrium, by an x-ray prior to the procedure. In patients who showed dysfunction of the LTCVC, a chest x-ray was performed to try to diagnose kinks. If positive, it was decided to replace the LTCVC by a TC or another LTCVC, situation where a new exit site and tunnel were created. If a kink was not diagnosed, the use of thrombolysis (alteplase) was attempted. In both cases, blood flow was improved, at least partially. There were 9 cases of kinks that required replacement of catheters, maintaining the same site.

A Brazilian group^8^ performed a similar study about LTCVC placement by TC- LTCVC conversion without fluoroscopy assistance. Infection was shown to be the major cause of catheter removal (14.5%), with an infection rate of 0.8/1000 catheter-day, in agreement with other studies that demonstrated a 1.0 - 1.1/catheter-day^5^^,^ 9 ^,^ 10. In our series, we had an infection rate of 1.87/catheter-day and infectious events was the second cause of catheter removal. Our high rate of infectious events was probably due to the low socioeconomic profile of the patients, which predisposes to a greater number of infectious episodes.

With respect to costs, our study showed that expenditures with this type of procedure were much lower when compared to those performed in private hospitals ($ 2,396 - value obtained through an average of budgets of private hospitals in the city of Natal) and in a US hospital ($ 4,885)^11^. Not using a hospital structure associated with the absence of patient sedation, a reduction in expenses was achieved without compromising patient safety. Implantation of a definitive catheter in a hospital is subjected to hospital rates (operating and recovery room, fluoroscopy assistance, hospital supplies), in addition to the medical fees and salaries of the nursing team.

Even considering the added costs of subjects who would need an additional fluoroscopy-guided procedure, the cost savings would remain substantial (taking into account the success rate presented). These costs coincide with the data presented by Becker et al^12^, who compared success rate, complications, and cost reduction with fluoroscopy-guided implant versus a technique similar to ours.

In addition to all the benefits already described, we can add the absence of radiation exposure by the nephrologist. Most interventional nephrologists spend a major portion of their work-time in the procedure room for a number of years and the effects of radiation are cumulative. Most dialysis patients require repetitive procedures usually guided by fluoroscopy. Therefore, the main goal of radiation management in interventional nephrology should be to minimize the unnecessary use of radiation^13^.

## Conclusions

In conclusion, the data generated from our study suggest that conversion of TC to LTCVC by a nephrologist in outpatient dialysis facilities do not carry higher risks of complications than those done in hospitals, and can serve as a safe alternative to centers that do not have fluoroscopy assistance available. In addition, the data show a substantial cost savings over placement of identical catheters by surgeons or radiologists in a hospital setting.
